# Reversal of Neuralgia Effect of Beta Carotene in Streptozotocin-Associated Diabetic Neuropathic Pain in Female Zebrafish via Matrix Metalloprotease-13 Inhibition

**DOI:** 10.3390/ph16020157

**Published:** 2023-01-22

**Authors:** Nallupillai Paramakrishnan, Laxmikant Chavan, Khian Giap Lim, Yamunna Paramaswaran, Arunachalam Muthuraman

**Affiliations:** 1 Department of Pharmacognosy, JSS College of Pharmacy, Mysore 643001, Karnataka, India; 2 Faculty of Medicine, AIMST University, Semeling, Bedong 08100, Kedah, Malaysia; 3 Pharmacology Unit, Faculty of Pharmacy, AIMST University, Semeling, Bedong 08100, Kedah, Malaysia

**Keywords:** fin clip test, matrix metalloprotease, palm oil mill effluent, pregabalin, reduced glutathione, thiobarbituric acid reactive substances

## Abstract

Beta carotene is a natural anti-oxidant agent, and it inhibits the matrix metalloprotease (MMP) activity. Diabetic neuropathic pain (DNP) is produced by cellular oxidative stress. The role of the beta carotene effect in diabetic neuropathic pain is not explored yet. The present study is designed for the evaluation of the palm oil mill effluent-derived beta carotene (PBC) effect in DNP in zebrafish. The DNP was induced by the intraperitoneal administration of streptozotocin (STZ). Blood glucose levels of above 15 mM were considered to be diabetic conditions. The zebrafish were exposed to test compound PBC (25, 50, and 100 µM), pregabalin (PG: 10 μM), and an MMP-13 inhibitor (CL-82198; 10 μM) for 10 consecutive days from day 11. The neuralgic behavioral parameters, i.e., temperature test, acetic acid test, and fin clip test were assessed on day 0 and the 7th, 14th, and 21st days. On the 22nd day, the blood glucose and MMP-13 levels and brain thiobarbituric acid reactive substances (TBARS), reduced glutathione (GSH), and MMP-13 activity levels were estimated. The treatment of PBC ameliorated the DNP-associated behavioral and biochemical changes. The results are similar to those of PG and CL-82198 treatments. Hence, the PBC possesses a potentially ameliorative effect against DNP due to its potential anti-oxidant, anti-lipid peroxidation, and MMP-13 inhibitory actions.

## 1. Introduction

Diabetes mellitus potentially causes damage to neuronal cells, which leads to them producing diabetic neuropathic pain (DNP). It involves pain sensations and numbness in the peripheral surface of the body [1,2]. Various experimental animal models have been used for the assessment of diabetic neuropathic pain such as rats and mice [3,4]. However, these models are more expensive and have various disadvantages [5]. Recently, zebrafish has become one of the acceptable animal models for pain assessment [6]. The zebrafish model also mimics the progression of human pain progression and sensations [7]. One of the major advantages of using zebrafish is that they feel pain with an indication of diving, erratic movements, and rubbing the injured area just such as we do [6]. In addition, they also respond to external stimuli such as thermal, pH changes, chemical irritants, and mechanical stimuli [8]. The progression of neuropathic pain is produced by potential cellular oxidative stress [9]. Even in diabetic conditions, the progression of neuropathic pain with cellular stress is faster [10]. The subsequent event of ion channel alteration, lipid peroxidation, the decline the endogenous anti-oxidant activity, mitochondrial dysfunctions, DNA fragmentation, and the expression of abnormal apoptotic proteins contribute to the progression of neuropathic pain and DNP [11,12,13]. Recently, the expression of major matrix metallopeptidases (MMP), i.e., MMP-12, and MMP-13, and their activities are identified in the progression of neuronal damage and degraded skin collagen, which leads to alterations in the healthy nerve–muscle–skin connections [14,15,16]. With everyday stresses and pathological diabetic conditions, the imbalance of the nerve–muscle–skin connection is very common [17]. The inhibitor of MMP-13 activity ameliorates the paclitaxel (it enhances the MMP-13 activity) treatment-associated neurotoxicity [15,18,19]. Further, pregabalin (PG) also performs neuropathic pain preventive action via the inhibition of the N-type calcium channel and MMP-9 activities [20]. Moreover, the recommended medications for diabetic neuropathic pain are anti-seizure drugs such as pregabalin, and gabapentin; antidepressant drugs, i.e., amitriptyline, nortriptyline and desipramine; serotonin and norepinephrine reuptake inhibitors (SNRIs), i.e., duloxetine and venlafaxine are widely used [21]. However, it produces various unwanted side effects such as drowsiness, dizziness, dry mouth, decreased appetite, constipation, orthostatic hypotension, and swelling in the hands and feet [22].

Beta carotene is one of the natural anti-oxidant agents, and it also acts as a vitamin A precursor. Experimentally, it is reported to inhibit MMP activity [23]. In diabetes conditions, the development of DNP is mainly due to the oxidative stress and associated activation of MMP-13 activity, which leads to produce glucose-mediated neurotoxicity and neuropathic pain [15]. Therefore, MMP-13 activity is a promising target for various inflammatory disorders such as osteoarthritis [24]. Hence, MMP-13 could be a target for the treatment of neuropathic pain disorders. However, the role of PBC action against the STZ-induced DNP and their MMP-13 inhibitory actions has not been explored yet. Hence, the present study is designed to investigate the role of PBC in the reversal action of the neuralgia effect in streptozotocin-associated DNP in female zebrafish models via matrix metalloprotease-13 inhibitory actions. In the present study, the selective MMP-13 inhibitor, i.e., CL-82198, and a non-selective MMP inhibitor and neuronal calcium channel blocker, i.e., PG, are used as reference drugs in this study.

## 2. Results

### 2.1. Estimation of Fasting Blood Glucose Levels

The administration of STZ (350 mg/kg; i.p.) significantly increased (*p* < 0.05) the fasting glucose levels compared to that which occurred in the normal control group. Moreover, the exposure to PBC (25, 50, and 100 µM) attenuated the STZ-induced elevated fasting blood glucose level in a dose-dependent manner. Moreover, the PBC doses, i.e., 50 and 100 µM, caused a more significant reduction of the fasting blood glucose levels than the 25 µM PBC treatment did. It indicates that the administration of PBC possesses a significant role in the regulation of the STZ-induced blood glucose levels, and this may be due to its anti-oxidant-mediated glandular protective actions. Moreover, the exposure to PG (10 μM), and CL-82198 (10 μM) also attenuated the STZ-induced elevated fasting blood glucose level. The results are tabulated in Table 1.

### 2.2. Assessment of DNP-Induced Changes in Thermal Pain Sensation

**Figure 1:**
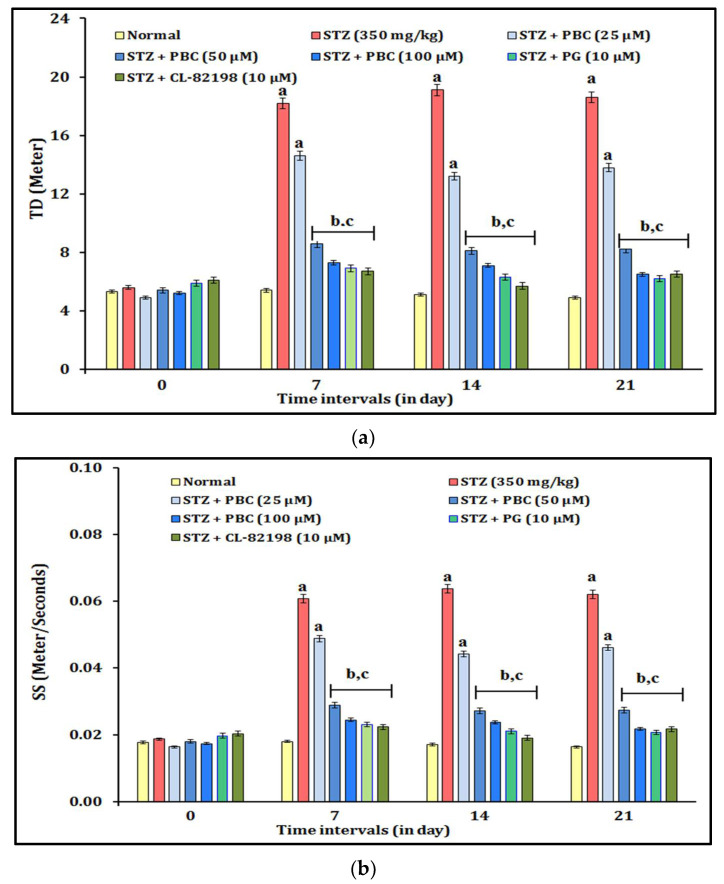
(**a**) Effect of PBC in DNP-induced TD changes in thermal pain sensation. Data are expressed as mean ± SD, n = 20 zebrafish per group. Abbreviation: CL-82198, MMP-13 inhibitor; PG, pregabalin; STZ, streptozotocin; TD, travel distance. (**b**) Effect of PBC in DNP-induced SS changes in thermal pain sensation. Data are expressed as mean ± SD, n = 20 zebrafish per group. Abbreviation: CL-82198, MMP-13 inhibitor; PG, pregabalin; SS, swimming speed; STZ, streptozotocin. ^a^ *p* < 0.05 vs. normal group. ^b^ *p* < 0.05 vs. STZ control group. ^c^ *p* < 0.05 vs. PBC (25 µM) group.

The DNP-induced changes in thermal pain sensation were assessed by the temperature test. The administration of STZ (350 mg/kg; i.p.) showed statistically significant (*p* < 0.05) changes in the thermal pain sensation in the temperature test experiment. The diabetic animals showed increased TD (travel distance) and SS (swimming speed) values. The alteration of the thermal pain sensation was observed at 0, 7, 14, and 21 days. Moreover, the exposure to PBC (25, 50, and 100 µM) showed an ameliorative effect against the STZ-induced thermal pain sensation changes in a dose-dependent manner. The PBC doses, i.e., 50 and 100 µM, were shown to have more significant effects on the reduction of the thermal pain sensation (i.e., TD and SS) than the 25 µM PBC treatment did. In addition, the exposure to the reference drugs, i.e., PG (10 μM) and CL-82198 (10 μM), also attenuated the STZ-induced thermal changes. It indicated that the exposure to PBC, PG, and CL-82198 shows their potential to ameliorate effect of the thermal pain sensation in zebrafish with DNP. The results of the PBC effects are illustrated in Figure 1a,b.

### 2.3. Assessment of DNP-Induced Changes in Chemical Pain Sensation

**Figure 2:**
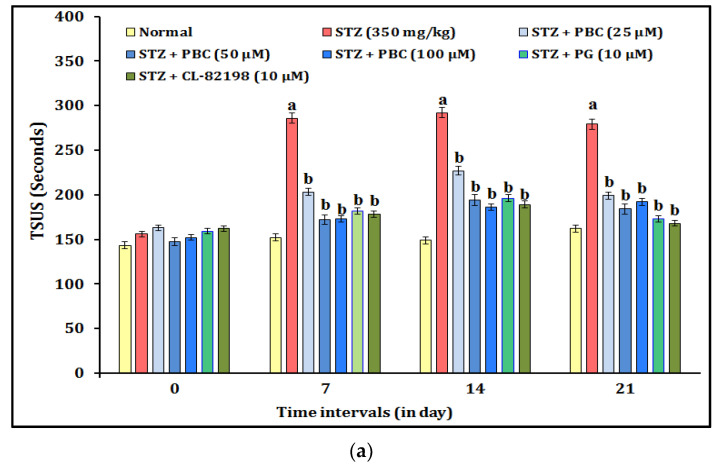

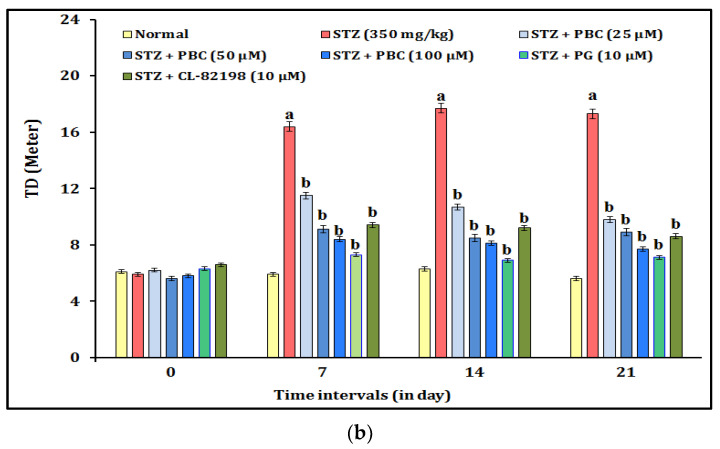
(**a**) Effect of PBC in DNP-induced TSUS changes in chemical pain sensation. Data are expressed as mean ± SD, n = 20 zebrafish per group. Abbreviation: CL-82198, MMP-13 inhibitor; PG, pregabalin; SS, swimming speed; STZ, streptozotocin; TSUS, time spent in the upper segment. (**b**) Effect of PBC in DNP-induced TD changes in chemical pain sensation. Data are expressed as mean ± SD, n = 20 zebrafish per group. Abbreviation: CL-82198, MMP-13 inhibitor; PG, pregabalin; SS, swimming speed; STZ, streptozotocin; TD, travel distance. ^a^ *p* < 0.05 vs. normal group. ^b^ *p* < 0.05 vs. STZ control group.

The DNP-induced changes in the chemical pain sensation were assessed by an acetic acid test. The administration of STZ (350 mg/kg; i.p.) showed statistically significant (*p* < 0.05) changes in the chemical pain sensation in the acetic acid test experiment. The diabetic animals showed increased values for TSUS (time spent in the upper segment) and TD (travel distance). The alteration of the chemical pain sensation was observed at 0, 7, 14, and 21 days. Moreover, the exposure to PBC (25, 50, and 100 µM) had an ameliorative effect against the STZ-induced chemical pain sensation changes in a dose-dependent manner. The PBC doses, i.e., 50 and 100 µM, did not have more significant effects on the reduction of the chemical pain sensation (i.e., TSUS and TD) than the 25 µM PBC treatment did. In addition, the exposure to the reference drugs, i.e., PG (10 μM) and CL-82198 (10 μM), also attenuated the STZ-induced above chemical pain changes. It indicated that the exposure to PBC, PG, and CL-82198 shows their potential to ameliorate the chemical pain sensation in the zebrafish with DNP. The results of the PBC effects are illustrated in Figure 2a,b.

### 2.4. Assessment of DNP-Induced Changes in Mechanical Pain Sensation

**Figure 3:**
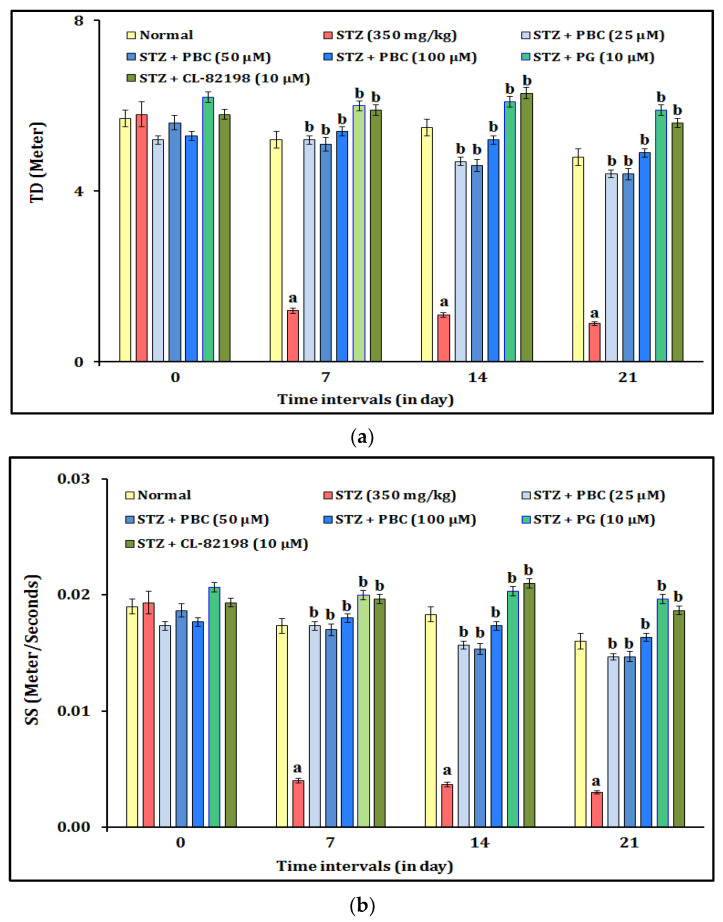
(**a**) Effect of PBC in DNP-induced TD changes in mechanical pain sensation. Data are expressed as mean ± SD, n = 20 zebrafish per group. Abbreviation: CL-82198, MMP-13 inhibitor; PG, pregabalin; SS, swimming speed; STZ, streptozotocin; TD, travel distance. (**b**) Effect of PBC in DNP-induced changes in mechanical pain sensation. Data are expressed as mean ± SD, n = 20 zebrafish per group. Abbreviation: CL-82198, MMP-13 inhibitor; PG, pregabalin; SS, swimming speed; STZ, streptozotocin; SS, swimming speed. ^a^ *p* < 0.05 vs. normal group. ^b^ *p* < 0.05 vs. STZ control group.

The DNP-induced changes in the mechanical pain sensation were assessed by a fin clip test. The administration of STZ (350 mg/kg; i.p.) showed statistically significant (*p* < 0.05) changes in the mechanical pain sensation in the fin clip test experiment. The diabetic animals showed decreased values for TD (travel distance) and SS (swimming speed). The alteration of the mechanical pain sensation was observed at 0, 7, 14, and 21 days. Moreover, the exposure to PBC (25, 50, and 100 µM) showed an ameliorative effect against the STZ-induced mechanical pain sensation changes in a dose-dependent manner. The PBC doses, i.e., 50 and 100 µM, did not have a more significant effect on the reduction of the mechanical pain sensation (i.e., TD and SS) than the 25 µM PBC treatment did. In addition, the exposure to the reference drugs, i.e., PG (10 μM) and CL-82198 (10 μM), also attenuated the STZ-induced mechanical pain changes. It indicated that the exposure to PBC, PG, and CL-82198 shows their potential to ameliorate the mechanical pain sensation in the zebrafish with DNP. The results of the PBC effects are illustrated in Figure 3a,b.

### 2.5. Estimation of DNP-Induced Biomarkers Changes

The administration of STZ (350 mg/kg; i.p.) produced a significant increase in the (*p* < 0.05) brain tissue TBARS and MMP-13 (also plasma) activity levels and decreases in the GSH level compared to those that occurred in the normal control group. The exposure to PBC (50 and 100 µM) attenuated the STZ-induced changes in the TBARS, MMP-13, and GSH activity levels in a dose-dependent manner. The exposure to PBC (25 µM) did not have a significant effect on STZ-induced changes in the TBARS, MMP-13, and GSH activity levels. It indicates that the administration of PBC (50 and 100 µM) possesses a significant role in the regulation of STZ-induced biomarkers changes due to its anti-oxidant, anti-lipid peroxidation, and regulation of collagenase-3 (MMP-13) functions. Moreover, of the exposure to PG (10 μM) and CL-82198 (10 μM) also attenuated the biomarker changes. The results are tabulated in Table 2.

## 3. Discussion

The result of the present study reveal that the intraperitoneal administration of STZ (350 mg/kg; i.p.) raised the fasting blood glucose levels above 15 mM, which indicates that the animals developed diabetes mellitus. In addition, STZ also increased the TD and SS in the thermal pain sensation test, raised the values of TSUS and TD in the chemical (acetic acid) pain sensation test, and decreased the values of TD and SS in the mechanical pain (fin clip) sensation test within the first 5 min of the test period. However, the exposure to PBC (25, 50, and 100 µM), PG (10 μM), and CL-82198 (10 μM) had the ameliorative effect on the STZ-induced biomarker changes. Biochemically, PBC also attenuated the STZ-induced changes in the abnormal fasting blood glucose and MMP-13 levels and the level of brain thiobarbituric acid reactive substances (TBARS), and it reduced the glutathione (GSH) and MMP-13 activity levels, which is similar to that which occurred in the reference drug (PG: 10 μM) and CL-82198 exposure.

The matrix metalloprotease-13 (MMP-13) is also called collagenase 3. It is a key enzyme for the cleavage of type II collagen, and its plays a vital role in the breakdown of cartilage in osteoarthritic conditions. In this condition, MMP-13 acts as a promising target [24]. Moreover, MMP-13 also plays a key role in the degradation of extracellular matrix proteins such as fibrillar collagen, fibronectin, total nucleated cells (TNC), and aggrecan core protein (ACAN) [25]. Further, MMP-13 also contributes to the progression of glucose-associated neurotoxicity via free radical generation [15], whereas the role of MMP-13 activity in the progression of DNP remains questionable; this is the major research question in this study. Another point of interest is PBC’s role in the regulation of MMP13 activity in DNP conditions. It has potentially pharmacological actions against various neurovascular disorders such as vascular dementia [26,27,28], Alzheimer’s disease, aging [29], and ischemic stroke [30]. Beta carotene acts as a vitamin A precursor, and it also possesses multiple cellular signaling actions such as those of free radical scavengers (anti-oxidant), it enhances the endogenous anti-oxidant activity level (raising the GSH synthesis), and it decreases the rate of the apoptosis (cell death) process, and it causes the inhibition of MMP. Therefore, PBC can ameliorate the STZ-induced DNP via MMP-13 inhibition. The result of this study evidences that PBC has MMP-13-inhibitory actions that are similar to those of the reference drugs of the MMP-13 inhibitor, i.e., CL-82198.

Moreover, beta carotene has neuromodulatory actions that occur via the acetylcholinesterase inhibitory actions [27]. It also possesses an anti-oxidant role in the prevention of neurovascular disorders [27,31] and reduces neurovascular complications [32]. In addition, carotenoids improve the quality of life in chronic pancreatitis and associated pain disorders via free radical scavenging actions [33]. The present study has also been shown to reduce the thermal, chemical, and mechanical pain sensations caused by STZ-induced glucose-mediated neurotoxicity. Clinically, it is proven that beta carotene reduces cellular oxidative stress via the improvement of glutathione synthesis and metabolism, and it enhances the endogenous anti-oxidant defense systems [34]. It also prevents the generation of reactive oxygen species (ROS) associated with lipid peroxidation in experimental animals and humans [35,36]. The progression of DNP behavior is also mediated through the elevation of ROS and lipid peroxidation [37]. The present results also reveal that DNP is ameliorated by PBC via the reduction of lipid peroxidation, and it enhances the GSH levels. Furthermore, the reference drug, i.e., PG, also ameliorates the DNP behavior via free radical scavenging, glutathione synthesis, and anti-lipid peroxidative actions. Experimentally, it is proven that PG attenuates the oxidative stress associated with fibromyalgia-like neuromuscular pain syndrome [38] and DNP [39]. It also possesses the inhibitory actions of MMPs, especially MMP-9 in vitro [20]. This is in agreement with our result. In the last few decades, it has been revealed that MMP is largely contributes to the progression of type of neuropathic pain, including DNP [16]. The strength of this research manuscript covers the simplest and most feasible methods of performing diabetic neuropathic pain assessments in zebrafish (lower vertebrate) animal models. Moreover, it provides more reliable and reproducible data of experiments on animals. However, the limitation of this study needs more technical handling skills for the collection of blood samples and the performance of zebrafish diabetic neuropathic pain behavior assessments. The research report revealed that the over-activation of MMP-9 and MMP-2 contributes to the progression of neuropathic pain. Further, the inhibitors of the above MMPs have prevented the various types of neuropathic pain, including DNP [16,40]. Recently, MMP-13 has been identified as a major contributor to glucose-associated neurotoxicity and chronic inflammatory disorders [15,24]. This is the first report that has revealed that the MMP-13 inhibitor, i.e., CL-82198, reverses the DNP-associated neuralgia effects.

## 4. Materials and Methods

### 4.1. Animals

Eight-month-old wild-type female adult zebrafish were used in the present study. The animals were kept in 10 L of potable drinking water in a housing tank. The tank conditions were maintained with aeration (for making the oxygen saturation), the temperature was maintained at 25 ± 0.5 °C with a thermostatic heating and cooling system for the water, and 14:10 h light and dark cycles were maintained throughout the experimental protocol. The zebrafish were allowed to acclimatize for 2 weeks. Thereafter, the experimental procedures were applied for the study of DNP in zebrafish animals. The pain behavior observations were carried out between 09.00 AM and 01.00 PM. This was essential to ensure the avoidance of hormonal influences in the assessment of neuralgic behaviors.

### 4.2. Drugs and Chemicals

Trichloro acetic acid, 5,5-dithibis(2-nitrobenzoic acid), reduced glutathione, thiobarbituric acid, 1,1,3,3-Tetra methoxy propane, and streptozotocin (STZ) were purchased from Sigma chemical, India. The palm oil mill effluents were collected from Palm Oil Mill Sdn. Bhd., Penang, Malaysia. The PBC was isolated by column chromatography techniques (Supplementary File). The reference control drug, i.e., PG, was procured from Glenmark Pharmaceuticals Limited Products, Mumbai, India. The selective MMP-13 inhibitor, i.e., CL-82198, was purchased from Abcam Inc. products, Chennai, India. The MMP-13 activity ELISA assay kit was obtained from Eagle Biosciences Inc., Nashua, NH, USA.

### 4.3. Induction of DNP

The diabetic condition of the zebrafish was produced by the intraperitoneal (i.p.) administration of STZ, as a described in a method by Wang et al. [41] and Rishitha and Muthuraman [42]. Briefly, all of the zebrafish were weighed under anesthetic conditions (in an ice-cold solution at 5 °C). The STZ (350 mg/kg; i.p.) was injected with 50 microliters of the stock solution using an insulin syringe (27 ½ gauge needle). The stock solution of STZ was prepared by thoroughly mixing 7 mg of STZ in 1 mL of normal saline (i.e., 0.9% *w/v* sodium chloride). The next day, the fasting (12 h) of the zebrafish was enacted for the collection of the blood drop samples. The blood drops were collected from zebrafish by performing gentle prick applications using a lancet needle on the side of the back fins of the zebrafish. The fasting blood glucose levels were estimated using a glucometer. A blood glucose level of above 15 mM glucose indicated that the zebrafish were diabetic. The diabetic zebrafish were kept for further 10 days in standard laboratory conditions for the progression and assessment of DNP behaviors.

### 4.4. Estimation of Plasma Glucose Levels

The plasma samples were used for the assessment of the glucose estimation, as described in the methods by Rishitha and Muthuraman [42] and Mohammadi et al. [43]. Briefly, the zebrafish were placed on the Petri dish, and a cross-section was made between the anal and caudal fins using a surgical blade. Then, the largest front portion of the zebrafish was immediately placed in a centrifuge tube (the tube was prefilled with 100 μL of anticoagulant solution, i.e., 11% *w/v* of sodium citrate) and vortexed at 1000 rpm for 10 min. Furthermore, the zebrafish were removed from the centrifuge tube, and the blood samples were centrifuged again at 2500 rpm for 15 min to obtain the plasma samples. The samples were diluted (1:4 ratio) with an anti-coagulant solution. The plasma samples were placed in glucometer for the estimation of the plasma glucose levels.

### 4.5. Experimental Protocol

Seven groups of adult female zebrafish (n = 20) were used in this research work. Group 1 served as a normal control group; Group 2 served as a diabetic neuropathic control group. This group was treated with STZ (350 mg/kg; i.p.) only. Groups 3, 4, and 5 served as the PBC exposure groups (25, 50, and 100 µM), and they were exposed for 10 consecutive days from day 11 [44,45]. Group 6 served as the PG exposure group (10 μM), and those in the group were exposed for 10 consecutive days from day 11 [46]. Group 7 served as the CL-82198 exposure group (10 μM), and those in the group were exposed for 10 consecutive days from day 11 [18]. All of the neuralgic behavioral parameters were assessed on days 0, 7, 14, and 21. On the 22nd day, the blood samples were collected as described in the method of Pedroso et al. [47] for the biochemical analysis, i.e., blood glucose and MMP-13. Thereafter, all of the zebrafish were sacrificed, and the brain samples were collected for the estimation of the TBARS, GSH, and MMP-13 activity levels.

### 4.6. Assessment of Neuralgic Behaviors in Zebrafish

The neuralgic behavioral changes in the zebrafish were assessed by multiple tests, i.e., a temperature test, an acetic acid test, and a fin clip test. The DNP behavioral assessments were carried out between 09.00 AM and 01.00 PM. The details of the thermal, chemical, and mechanical pain sensations in the adult female zebrafish are described in the following sections.

#### 4.6.1. Temperature Test

A temperature test is one of the common thermal methods used for the assessment of pain sensations, as described by Ohnesorge et al. [48]. Briefly, the dimensions of the experimental test apparatus are 15 cm in diameter and 08 cm in height, and a flat transparent circular pyrex glass jar was used. The tanks were covered with an opaque layer sheet to prevent the influence of external visual stimuli. The water level was maintained up to the level of 05 cm, and the bottom part was placed on grid stickers. The jar was kept on a thermos-controlled (48 °C) hot plate surface. Normally, zebrafish are characterized by wide thermal tolerance of between 6.7 and 41.7 °C [49]. The neuralgic effect was evaluated by the assessment of “Travel Distance”, termed TD, and “Swimming Speed” (SS). Generally, when an animal is placed in a new chamber, it prefers the move faster than it normally does. Hence, the animal was allowed exposed to the jar for 2 min (acclimatization period). If the animal’s mouth hit the jar’s wall, we took it off the plate for 10 s and allowed it to have an additional 1 min swim in the jar portions. The total observation times for all of the fish’s responses were fixed to a 5 min period. A long TD and rapid SS movement of the zebrafish are known to suggest that the fish is experiencing severe thermal neuropathic pain sensations.

#### 4.6.2. Acetic Acid Test

The acetic acid test is one of the common chemical methods used for the assessment of pain sensations, as described by Taylor et al. [50]. Briefly, 5% acetic acid was injected into muscular tissue between the dorsal and caudal fins. The chemical pain sensations were assessed by using vertical glass jar apparatus. The glass jar was 20 cm in length, 6 cm in width, and 20 cm in height. The jar was filled with 18 cm of water, and it was divided into three equal horizontal portions with external markings. Three sides of the wall and bottom surface of the jar were covered with an opaque layer sheet to prevent the influence of external visual stimuli. The front side remained transparent (with grid lining) for the video recording of the fish’s movements. Movements suggestive of pain by the zebrafish were assessed as with the indicators of “Time spent in the upper segment” (TSUS) and “Travel distance” (TD). Generally, when an animal is placed in a new chamber, it prefers the swim to multiple segments. Hence, animal was exposed to the jar for 2 min (acclimatization period). The total observation time for all of the fish’s responses was fixed to 5 min period. Raised TSUS and TD movement levels of zebrafish are known to suggest that they are experiencing severe chemical neuropathic pain sensations.

#### 4.6.3. Fin Clip Test

The fin clip test is one of the common mechanical methods used for the assessment of pain sensations, as described by Deakin et al. [51]. Briefly, the zebrafish were anesthetized with an ice-cold solution (5 °C) until we observed slowness of the gill movements (2–3 min). The anesthetized zebrafish were transferred to a Petri dish using a plastic spoon. The dorsal fin was loaded with an aluminum foil clip (50 mg, 6 mm length, and 10 mm width folded) with a slight modification (see Dou et al. [52]). The mechanical pain sensations were assessed by using the vertical glass jar apparatus. The glass jar was 20 cm in length, 6 cm in width, and 20 cm in height. The jar was filled with 18 cm of water. Three sides of the wall and bottom surface of the jar were covered with an opaque layer sheet to prevent the influence of external visual stimuli. The front side remained transparent (with grid lining) for the video recording of the fish’s movements. Painful movements by the zebrafish were assessed using the indicators of “Travel distance” (TD) and “swimming speed” (SS). Generally, when an animal is placed in a new chamber, it prefers the swim to multiple segments. Hence, the animal was exposed to the jar for a period of 1 min (acclimatization period). The total observation time for all of the fish’s responses were fixed for 5 min. The reduction of the TD and SS levels of the zebrafish are known infer severe mechanical neuropathic pain sensations.

### 4.7. Brain Sample Collection

After the assessment of neuralgic behavioral changes in the zebrafish, the brain samples were collected using microsurgical techniques under a stereomicroscope. Then, the samples were homogenated with a phosphate buffer (pH 7.4) solution. The solutions were centrifuged at a force of 1372× *g* for 15 min. The supernatant was collected and stored in a deep freezer at −4 °C for further a biochemical estimation. The tissue biomarker changes, i.e., thiobarbituric acid reactive substances (TBARS), reduced glutathione (GSH), and MMP-13 activity levels were estimated in these samples.

#### 4.7.1. Estimation of TBARS Level

The elevation of the TBARS level is an indicator of lipid peroxidation. The levels of TBARS in zebrafish brain samples were estimated by the spectroscopic method, as described in the method of Ohkawa et al. [27], using minor modifications by Muthuraman and Rishitha [53]. Briefly, 0.2 mL of tissue supernatant was thoroughly mixed with 0.2 mL of 8.1% *w/v* of sodium dodecyl sulphate, 1.5 mL of 30% *v/v* of acetic acid (pH 3.5), and 1.5 mL of 0.8% *w/v* of thiobarbituric acid. The tube volume was made up of 4 mL of distilled water. Then, the test tubes were incubated at 95 °C for 1 h. Furthermore, the tubes were cooled with tab water, which was applied to the outer surface of the test tubes. Crucially, 1 mL of distilled water and 5 mL of n-butanol-pyridine (15:1 *v*/*v*) mixture were added to each test tube. Then, the tubes were centrifuged at 1372× *g* force for 15 min. The clear pink color chromogen was formed, and its intensity is based on the presence of TBARS contents in the tissue samples. The changes in the absorbance were recorded at the 535 nm wavelength using a spectrophotometer (DU 640B Spectrophotometer, Beckman Coulter Inc., Pasadena, CA, USA). The standard plot of TBARS was prepared with 1–10 nM of 1,1,3,3-tetra methoxy propane. The TBARS levels in the tissue samples were obtained by performing a further calculation with the correlation of the tissue protein contents. The TBARS results are expressed as nM per mg of protein.

#### 4.7.2. Estimation of GSH Level

A reduced level of GSH is an indicator of oxidative stress in the tissue. The GSH levels in the zebrafish brain samples were estimated by the spectroscopic method, as described in the method of Ellman [28], with minor modifications by Muthuraman and Rishitha [53]. Briefly, 0.5 mL tissue supernatant was mixed with 2 mL of 0.3 M of disodium hydrogen phosphate solution and 0.25 mL or 0.001 M of DTNB solution. The clear yellow color chromogen developed during the incubation of the test tubes at room temperature for 5 min. The changes in the yellow color chromogen intensity levels indicate the presence of the GSH content in the tissue sample. The changes in the absorbances were recorded at the 412 nm wavelength by using a spectrophotometer (DU 640B Spectrophotometer, Beckman Coulter Inc., Pasadena, CA, USA). The standard plot of GSH was prepared with 5–50 µM of the reduced form of glutathione. The GSH levels in the tissue samples were obtained by performing a further calculation with the correlation of the tissue protein contents. The results are expressed as μM of GSH/mg of protein.

#### 4.7.3. Estimation of Total Protein Level

The total protein levels were estimated using the spectroscopic method, as described in the method of Lowry et al. [54], with minor modifications by Muthuraman and Rishitha [53]. Briefly, 300 μL of zebrafish brain supernatants was diluted with 1 mL of distilled water. About 5 mL of Lowry’s reagents was mixed with a supernatant solution and incubated at room temperature (37 °C) for further 15 min. Thereafter, 0.5 mL of Folin–Ciocalteu reagent was added and vortexed vigorously at room temperature (37 °C) for 30 min. The formation of an intense, clear purple chromogen indicates the presence of protein contents in the tissue sample. The changes in the absorbance were recorded at the 750 nm wavelength by using a spectrophotometer (DU 640B, UV-Spectrophotometer, Beckman Coulter Inc., CA, USA). The standard plot of the total protein was prepared with 1–10 mg/mL of bovine serum albumin. The total protein levels in the tissue samples were obtained by performing a further calculation of other tissue biomarker contents. The results are expressed as mg of protein per ml of supernatant.

#### 4.7.4. Estimation of MMP-13 Activity Assay

MMP-13 activity is involved in the breakdown of the extracellular matrix in normal physiological processes. However, the over-activation of MMP-13 is known to cause tissue damage and oxidative stress-mediated neurotoxicity [15]. In pathological inflammatory conditions, the MMP-13 levels are elevated in the blood, synovial fluid, and tissue supernatants. The MMP-13 levels were analyzed by using the Enzyme-linked immunosorbent assay (ELISA) kit method. The blood samples were collected from the zebrafish using a method described by Babaei et al. [55], and the brain samples were collected using a method described by Vargas et al. [56,57]. The samples (50 µL plasma and 100 µL tissue supernatant) from the zebrafish were used for the assessment of the MMP-13 activity. Briefly, the samples were placed in a monoclonal antibody pre-coated microplate and incubated at room temperature for 10 min. Then, the microplate was washed, and an MMP activator was added, *p*-aminophenylmercuric acetate (APMA), and it was incubated at room temperature for 10 min, then we washed the microplate with a washing buffer. Finally, the fluorogenic substrate was added (the cleavage site of MMP-13 is Gly-Leu), and it was incubated at room temperature. The reaction was stopped with a stopping solution. The fluorescence intensity was determined at the excitation/emission wavelength of 320 nm/405 nm. The MMP-13 activity is expressed as nanogram per milliliter (ng/mL) using a microplate reader (ClaIR™, Photon Etc., Montreal, Canada). The standard plot of the MMP-13 activity was prepared using 0, 32, 63, 125, 250, 500, 1000, and 2000 pg/mL of it. The results were expressed as ng/mL of the MMP-13 activity.

### 4.8. Statistical Analysis

All of the results are expressed as mean ± standard deviation (SD). The data obtained from behavioral tests were statistically analyzed using one-way analysis of variance (ANOVA), followed by Tukey’s test, which was applied by using Graph pad prism Version 5.0 software (Science Plus Group BV, Groningen, The Netherlands). The data of biomarkers, i.e., TBARS, GSH, and MMP-13 activity levels were analyzed using one-way ANOVA, followed by Tukey’s multiple range tests, and we applied a Post hoc analysis by using Graph pad prism Version 5.0 software. A probability value of *p* < 0.05 (alpha = 0.05; 95% confidence interval) was considered to be statistically significant.

## 5. Conclusions

The exposure to PBC, PG, and CL-82198 reverses the STZ-induced DNP behavior in female zebrafish. Hence, PBC possesses the ability to reverse STZ-associated DNP in female zebrafish via its potential blood glucose regulation, anti-oxidant, anti-lipid peroxidation, and MMP-13 inhibitory actions.

## Figures and Tables

**Table 1:** Effect of PBC in STZ-induced fasting blood glucose levels.

Groups	Fasting Blood Glucose Level (mg/dL)
Normal	72.3 ± 1.6
STZ (350 mg/kg)	141.2 ± 2.1 ^a^
STZ + PBC (25 µM)	101.8 ± 2.4 ^b^
STZ + PBC (50 µM)	83.5 ± 2.2 ^b,c^
STZ + PBC (100 µM)	76.1 ± 1.2 ^b,c^
STZ + PG (10 μM)	74.5 ± 1.3 ^b,c^
STZ + CL-82198 (10 μM)	79.4 ± 1.6 ^b,c^

Data are expressed as mean ± SD, n = 20 zebrafish per group. ^a^ *p* < 0.05 vs. normal group. ^b^ *p* < 0.05 vs. STZ control group. ^c^ *p* < 0.05 vs. PBC (25 µM) group. Abbreviation: CL-82198, MMP-13 inhibitor; PG, pregabalin; STZ, streptozotocin.

**Table 2:** Effect of PBC in STZ-induced biomarker changes.

Groups	Tissue GSH (μM/mg of Protein)	Tissue TBARS (nM/mg of Protein)	Tissue MMP-13 (ng/mL)	Plasma MMP-13 (ng/mL)
Normal	6.52 ± 0.04	0.16 ± 0.009	0.46 ± 0.13	0.06 ± 0.08
STZ (350 mg/kg)	1.03 ± 0.11 ^a^	1.95 ± 0.004 ^a^	1.74 ± 0.18 ^a^	1.14 ± 0.04 ^a^
PBC (25 µM)	3.92 ± 0.09 ^a^	0.97 ± 0.008 ^a^	1.35 ± 0.14 ^a^	0.89 ± 0.09 ^a^
PBC (50 µM)	4.63 ± 0.07 ^b,c^	0.56 ± 0.005 ^b,c^	0.68 ± 0.12 ^b,c^	0.37 ± 0.05 ^b,c^
PBC (100 µM)	5.75 ± 0.11 ^b,c^	0.32 ± 0.007 ^b,c^	0.55 ± 0.08 ^b,c^	0.33 ± 0.04 ^b,c^
PG (10 μM)	5.43 ± 0.08 ^b,c^	0.29 ± 0.012 ^b,c^	0.49 ± 0.11 ^b,c^	0.25 ± 0.06 ^b,c^
CL-82198 (10 μM)	5.96 ± 0.03 ^b,c^	0.18 ± 0.011 ^b,c^	0.47 ± 0.09 ^b,c^	0.14 ± 0.03 ^b,c^

Data are expressed as mean ± SD, n = 20 zebrafish per group. ^a^ *p* < 0.05 vs. normal group. ^b^ *p* < 0.05 vs. STZ control group. ^c^ *p* < 0.05 vs. PBC (25 µM) group. Abbreviation: CL-82198, MMP-13 inhibitor; MMP-13, matrix metallopeptidase-13; PG, pregabalin; GSH, reduced glutathione; STZ, streptozotocin; TBARS, thiobarbituric acid reactive substances.

## Data Availability

Data Availability Statement: Data is contained within the article and supplementary material.

## Supplementary Materials

The following supporting information can be downloaded at: https://www.mdpi.com/article/10.3390/ph16020157/s1.
