# Supplementary material for: Reversal of Neuralgia Effect of Beta Carotene in Streptozotocin-Associated Diabetic Neuropathic Pain in Female Zebrafish via Matrix Metalloprotease-13 Inhibition
Source: Pharmaceuticals (Basel). 2023 Jan 22;16(2):157. doi: 10.3390/ph16020157 (PMC9959792; doi:10.3390/ph16020157)
Supplement: Supplementary file 1 [file pharmaceuticals-16-00157-s001.zip › pharmaceuticals-2108654-supplementary.pdf]

Isolation of beta carotene by column chromatography techniques

The palm oil mill effluent-derived beta carotene (PBC) was isolated by column chromatography as a described method by Amalya and Sumathy [1] and Wingqvist [2]. Briefly, the palm oil mill effluent was concentrated by using a rota-evaporator (RE601 Series, Yamato Scientific Co. Ltd, Tokyo, Japan) and a water bath (IBATA scientific technology, Tokyo, Japan). The separation of the compound was made making the column with mobile and stationary phases. The stationary phase was placed in the column (25 cm long & 4 to 5 mm in internal diameter containing 5 μm packing materials) chromatography. This standard column was filled with adsorbent, cotton, and silica gel. The mobile phase was used by multiple solvents *i.e.*, acetone, hexane, and distilled water. The flow rates of mobile solvent for column chromatography were maintained at 1 to 2 ml per minute. About 10 grams of palm oil mill effluent was weighed and mixed with acetone and transferred into the centrifuge tube. Thereafter, 5 ml of hexane was further added and shaken completely followed by 5 ml of distilled water mixed and shaken well with an occasional opening (venting). Now, the tube has a mixture of PBC in the emulsion form. The mixture was centrifuged at 4000 rpm for 20 minutes by an ultracentrifugation machine (Thermo Fisher Scientific, Selangor, Malaysia) to break the emulsion of PBC. The pigment layer appeared in the hexane layer (top) and it was dark green. However, acetone was dissolved in the water. The dark green pigment layer was carefully separated and dried by adding a solution of 0.5 grams of anhydrous sodium sulfate. After 5 minutes the PBC solution was used for the further separation of PBC by column chromatographic method.

The silica gel (15 grams) was mixed with 100% hexane solution and this slurry-like paste was filled in a column chromatography device without air trapping. After 1 hour, the solvent was drained until the 1cm level of silica gel packing. Then, the PBC solution was poured into the column and drained the solvent at a constant flow rate (1 ml per minute) via silica gel column packing. The reddish-yellow coloured beta-carotene was separated with further elution with 100% hexane. Throughout the process, the column was covered with aluminum foil due to the prevention of a light-sensitive reaction of beta-carotene. Moreover, the fractions were collected and evaporated to get the concentrated carotene. The total bata-carotene content was calculated by the spectrophotometric method by measurement of absorbance changes at 450 nm using a spectrophotometer (DU 640B Spectrophotometer, Beckman Coulter Inc., CA, USA). Thereafter, the beta carotene was quantified by using the following formula:

$$\text{Total beta carotene}\left(\frac{\mu g}{g}\right) = \text{A x V (ml) x}\ \frac{10^4}{A^{1\%}\ \text{cm}}\ \text{x W (g)}$$

Where, A represents the absorbance of the beta-carotene pigment at 450 nm; V represents the total extract volume; $A^{1\%}$cm represents the absorption coefficient of β carotene in hexane; W represents the sample weight.

References

1. Amalya, J.T.; Sumathy, J.H. Analysis of Carotenoid Pigments Extracted By Column Chromatography From the Leaves and Flowers of Peltophorum Pterocarpum By Thin Layer Chromatography , Mass

Spectrometry and Nuclear Magnetic Resonance Spectroscopy. In Proceedings of the Unique Research Journal of Chemistry; 2014; Vol. 2, pp. 11–17.
2. Wingqvist, A. Extraction, Isolation and Purification of β-Carotene 2011.
